# Not All U.S. Pharmacists Are Equal: A Full-Time Versus Part-Time Comparison

**DOI:** 10.3390/pharmacy13050149

**Published:** 2025-10-17

**Authors:** Ioana Popovici, Manuel J. Carvajal

**Affiliations:** Department of Sociobehavioral and Administrative Pharmacy, Barry and Judy Silverman College of Pharmacy, Nova Southeastern University, 3200 South University Drive, Fort Lauderdale, FL 33328-2018, USA; cmanuel@nova.edu

**Keywords:** earnings, gender, human capital, income gaps, job-related preferences, labor input, part-time employment, pharmacist workforce

## Abstract

Part-time employment is an increasingly important feature of the U.S. labor market, yet little is known about how earnings determinants differ between full-time and part-time pharmacists. Few prior studies have compared earnings models across these groups, but most have relied on small or geographically limited samples. Moreover, the dynamic and rapidly evolving nature of the labor market makes this study especially timely, as most prior research on pharmacist earnings is based on older data. This study examined earnings determination separately for full-time and part-time pharmacists, estimating the influence of work input, human capital, demographic characteristics, and job-related features within each group. Data were obtained from the 2019–2022 American Community Survey (ACS), a large, continuous, nationally representative survey conducted annually by the U.S. Census Bureau. The sample included 12,064 pharmacists (4667 men and 7397 women) aged 25–64 years, practicing in the U.S. Ordinary least-squares equations were estimated separately for male and female pharmacists within each employment category, allowing comparison of the direction, magnitude, and statistical significance of covariates across groups. Results revealed notable differences in the earnings effects of several factors between full-time and part-time pharmacists, highlighting the interaction of individual choices and structural market forces in shaping compensation. These findings can inform workforce planning and guide the development of targeted job-related incentives to support retention and satisfaction across employment types.

## 1. Introduction

Part-time employment is a complex and increasingly prominent component of labor markets, shaped by workplace opportunities and constraints, the dynamics of labor demand and supply, and the diverse needs and preferences of workers [1,2]. On the labor demand side, employers offer part-time positions to accommodate seasonal and cyclical fluctuations in consumer demand, provide services during non-standard hours, cover gaps in full-time staffing, and reduce operational costs through lower wages and fewer benefits [3,4]. On the labor supply side, part-time work allows individuals to combine professional obligations with non-work activities, pursue work–life balance, or remain employed when full-time work is not feasible [5,6,7,8]. While some part-time arrangements are involuntary, driven by economic necessity, many are voluntary, reflecting conscious choices to trade off income for flexibility, leisure, or personal and family obligations [2,9,10,11,12].

The last few decades have witnessed substantial growth in part-time employment across industrialized countries [13,14], including in health-related professions such as pharmacy [15,16]. In the U.S. pharmacists represent a critical component of the healthcare workforce. The U.S. pharmacist workforce has grown by nearly 18% from 2018 to 2023, with women now comprising about 60–62% of pharmacists. Employment is concentrated in community (≈53%) and hospital (≈24%) settings, with smaller proportions in other sectors [17]. While most pharmacists work full-time, roughly 15% work part-time, and women are more than twice as likely as men to do so. Full-time pharmacists typically work about 44 h per week for 48–49 weeks annually. Although the overall supply of pharmacists has kept pace with national demand, regional shortages persist, particularly in rural and underserved areas. At the same time, recruitment and retention challenges have emerged as employment opportunities diversify across settings such as hospitals, clinics, and mail-order services. The profession also is marked by a strong gender imbalance, with women disproportionately represented in part-time positions, raising questions about equity, career progression, and the long-term stability of the workforce. Unlike many occupations, pharmacy offers relatively linear remuneration with respect to hours worked and comparatively low penalties for reduced work input [18,19]. Consequently, much of the part-time work observed among pharmacists is expected to be voluntary, motivated primarily by the higher opportunity costs of non-work activities [20]. Nonetheless, part-time pharmacists may face disadvantages beyond lower earnings, including slower promotion trajectories, reduced access to leadership roles, and perceptions of lower commitment compared with full-time colleagues [21].

Prior evidence suggests that part-time pharmacists differ from their full-time peers not only in hours worked but also in how earnings are determined. Pharmacists working fewer hours tend to focus primarily on pay, whereas full-time pharmacists often consider a broader set of factors, including professional development and job satisfaction [15]. These distinctions raise important questions about whether part-time pharmacists are a subset of a uniform group of pharmacists or vary substantially in characteristics and earnings responses both within their group and relative to full-time workers.

The purpose of this study was to examine, separately for full-time and part-time pharmacists, the income determination process in the pharmacist workforce by estimating a model depicting earnings as a function of work input, human capital, demographic characteristics, and job-related features. Few studies compare earning determination models for full- and part-time pharmacists, and most of these use limited data sets or small, outdated samples. This study used recent data from the American Community Survey, a large, nationwide survey conducted annually by the U.S. Census Bureau. By comparing these responses across employment categories, the study aimed to illuminate how professional compensation is shaped by individual choices and structural factors in the pharmacist labor market and identify ways in which job-related incentives might be tailored to each group.

Employment status influences not only earnings but also career trajectories, ownership opportunities, and the long-term sustainability of the workforce. Differences in how men and women, single parents, or minority pharmacists participate in the labor market may point to structural inequities that influence professional advancement and retention. Moreover, shifts in the balance of full-time versus part-time work may affect the availability of pharmacy services in communities, particularly in retail settings where patient access is closely tied to staffing. By examining these dynamics, this study was designed to contribute to workforce policy discussions and help identify factors that shape both the equity and efficiency of the pharmacy profession.

## 2. Methods

The income determination model developed in this study was applied to full-time and part-time pharmacists practicing in the U.S. Full-time pharmacists were defined as working an average of at least 30 h per week throughout the year; pharmacists working fewer than 30 h per week, on average, were considered part-time. Separate equations with identical covariates were specified, estimated using ordinary least-squares, and tested for statistical significance for male and female pharmacists within each employment category, resulting in a total of four equations. (Ordinary least-squares is a statistical procedure that minimizes the sums of squares of the error terms; it provides the best-fit estimates for the covariates. Statistical significance at 0.01 and 0.05 levels were used). Since the covariates were consistent across equations, the estimated coefficients were utilized to compare the direction, magnitude, and significance of each covariate’s effect on earnings while controlling for all other factors.

### 2.1. Data Source

The data were drawn from the 2019–2022 American Community Survey (ACS), which is conducted by the U.S. Census Bureau [22]. Utilization of recent data is particularly important given the continuous evolution of the labor market and the potential for shifts in work patterns, compensation structures, and employment preferences over time. Every year over 3.5 million households are invited by the U.S. Census Bureau to provide, anonymously, data on social, economic, labor force, and other variables as part of the ACS. The data are gathered every month from a rotating sample of about 250,000 addresses in the 50 states, Washington, D.C., and Puerto Rico.

Pharmacists were selected from the global survey utilizing the 2010 ACS occupation classification scheme. Only pharmacists between the ages of 25 and 64 years, who worked at least 100 h and earned an average of at least $10.00 per hour during the previous year, were included. (These parameters were set to weed out observations containing misreported data.) The current sample included 12,064 pharmacists (4667 men and 7397 women) working in the U.S. The Consumer Price Index (CPI) [23] was used to adjust incomes for inflation to 2022 dollars.

This research was supported entirely by internal university funds. Institutional Review Board’s assessment was not necessary as the data were deidentified and publicly accessible.

### 2.2. Variables in the Model

The model interpreted, for each gender within each employment category, pharmacists’ earnings as a function of work input, human capital, demographic features, and job characteristics. Specifically,

ln E_ijk_ = α_ij_ + W_hijk_λ_hij_ + D_hijk_γ_hij_ + X_hijk_θ_hij_ + Z_ijk_φ_ij_ + u_ijk_

where

ln E*_ijk_* is a vector of the natural logarithm value of annual income reportedly earned by the *k*th pharmacist of the *j*th gender in the *i*th employment category;

W*_hijk_* is a matrix of two sets of work input values, number of hours worked per year, measured in 100 h units (*h* = 1), and number of hours worked per year squared, also measured in 100 h units (*h* = 2), reported by the *k*th pharmacist of the *j*th gender in the *i*th employment category;

D*_hijk_* is a matrix of human capital characteristics, including age (*h* = 1) and age squared (*h* = 2), reported by the *k*th pharmacist of the *j*th gender in the *i*th employment category;

X*_hijk_* is a matrix of three sets of demographic-features values, including dichotomous variables for currently not being married (*h* = 1), being Black/African American (*h* = 2), and being Hispanic (*h* = 3), reported by the *k*th pharmacist of the *j*th gender in the *i*th employment category;

Z*_ijk_* is a vector for job characteristics, consisting of a dichotomous variable for working in retail pharmacy, reported by the *k*th pharmacist of the *j*th gender in the *i*th employment category;

u*_ijk_* is a vector of normally and independently distributed stochastic disturbance terms, with mean zero and variance σ*_ij_*^2^, pertaining to the *k*th pharmacist of the *j*th gender in the *i*th employment category;

α*_ij_* is the least-squares constant term for the *j*th gender in the *i*th employment category;

λ*_hij_* is a vector of two work-input parameters estimated for the *j*th gender in the *i*th employment category;

γ*_hij_* is a vector of two age parameters estimated for the *j*th gender in the *i*th employment category;

θ*_hij_* is a vector of three demographic-features parameters estimated for the *j*th gender in the *i*th employment category;

φ*_ij_* is a retail pharmacy coefficient estimated for the *j*th gender in the *i*th employment category;

and where

*i* = 1 for full-time pharmacists, and *i* = 2 for part-time pharmacists;

*j* = 1 for male pharmacists, and *j* = 2 for female pharmacists;

*k* = 1, …, n*_ij_*; and

n*_ij_* is the number of pharmacists of the *j*th gender in the *i*th employment category.

The dependent variable was pharmacists’ annual, pre-tax, wage-and-salary plus self-employment income earned. This was adjusted for inflation to 2022 dollars using the Consumer Price Index (CPI). Income was logged to control for the influence of outliers, in such a way that the estimated coefficients depicted relative differences rather than absolute amounts. (At any level of logged income, an addition of 0.693147 units to the value of the dependent variable doubled earnings, while a subtraction of 0.693147 units from the dependent variable cut earnings in half.)

Intuitively, work input is the most important determinant of labor-related income. Goldin and Katz [18] even have suggested that in pharmacy it is the only relevant variable. Work input was measured in 100 h units to facilitate interpretation, so that each coefficient reflects the estimated effect of an additional 100 h of work per year. Linear and quadratic components were identified in the equations; the quadratic component was included to account for diminishing returns. The linear terms were expected to be positive, and the quadratic terms were expected to be negative.

Age, commonly treated as an indicator of experience [19], was chosen to measure skills and experience imbedded in pharmacists’ human-capital stock. It also appeared in the model with a linear and a quadratic component. More human capital was anticipated to reflect greater productivity and more income up to a point beyond which obsolescence would occur; consequently, positive linear terms and negative quadratic terms were expected [24].

The demographic-features matrix consisted of three dichotomous covariates. The first was assigned a value of 1 if the pharmacist was not married (i.e., single, separated, divorced, or widowed) to measure the penalty of not being married [25], so the estimated coefficients were anticipated to be negative. This variable was included as a parameter of interest because prior research in labor economics and health workforce studies has shown that marital status can influence earnings through multiple channels [2,5]. For example, married individuals may have different work–life arrangements, spousal support, or household responsibilities that affect labor supply, job mobility, or negotiation opportunities, all of which can impact earnings. The other two demographic-features covariates identified Blacks/African American and Hispanic minorities. Several studies have concluded that belonging to either or both minority groups is associated with negative labor outcomes [26,27,28]. Therefore, the least-squares coefficients of both covariates were hypothesized to be negative, suggesting relatively lower earnings for minority pharmacists.

The job characteristic chosen for this study was a dichotomous variable indicating that the pharmacist worked in retail pharmacy. This setting typically involves a high volume of medication dispensing, which often is perceived as less desirable and more stressful than other environments, such as hospital pharmacy, but is generally associated with higher pay [29]. Consequently, the coefficients were expected to be positive. Working in retail pharmacy was a variable constructed based on a 4-digit variable reporting the type of industry in which the person performed an occupation based on the North American Industrial Classification System.

Beyond the covariates discussed here, the influence on income of other covariates was tested. These covariates, which also appear frequently in the literature, measured being “White” and “other races” as part of ethnic identification, “number of children in the household” and “having at least one child” as family composition, being “single/never married” and “separated/divorced/widowed” as part of marital status, and working in “hospital pharmacy” and “manufacturing or other” as well as “self-employed” as part of job characteristics. None of these covariates yielded consistently significant coefficients. Their contribution to explaining full-time and part-time pharmacists’ earnings was so feeble that the least-squares coefficients of the other covariates appearing in the reported equations did not change appreciably when they were deleted. Thus, they were excluded.

## 3. Results

The results indicated that 11.3% of pharmacists in the sample worked part-time and 61.3% were women. The gender composition varied widely by employment category—14.6% of women and 6.0% of men worked part-time.

### 3.1. Employment Category and Gender Composition

The means and standard deviations of income earned by respondents, as well as variables postulated to affect them, are presented by occupational category and gender in Table 1 (The *t* statistic with uneven observations was used to compare gender values within employment category and employment-category values within gender.). Full-time men earned higher income levels than full-time women, but part-time women earned higher income levels than part-time men. Similarly, full-time men worked on average more hours per year than full-time women, but part-time women worked more hours than part-time men.

Male pharmacists who worked full-time were older than their female counterparts, but no significant gender differences in age was detected among part-time pharmacists. Part-time female pharmacists were older than full-time female pharmacists, but no significant difference by employment category seemed to exist among male pharmacists.

Less than one-third (29.3%) of all pharmacists reported being single, separated, divorced, or widowed, and significant variations by employment category and gender were detected. Among full-time pharmacists, the percentage of non-married women exceeded the percentage of non-married men, but among part-time pharmacists the percentage of non-married men exceeded the percentage of non-married women. Among men, proportionately more part-time than full-time pharmacists were not married, but the opposite was true among women—more full-time than part-time pharmacists were not married.

Only 4.9% of pharmacists in the sample were Black/African American, and they were unevenly distributed by employment category and gender. There were proportionately more Black/African American women than men among full-time pharmacists, but Black/African American male part-time pharmacists proportionately exceeded Black/African American female part-time pharmacists. Among women there were proportionately more Black/African American full-time than part-time pharmacists, but among men there was no significant difference by employment category. Hispanics, the other minority whose effect on income was measured here, also accounted for a small fraction (4.3%) of the pharmacist workforce, but unlike Black/African Americans, they showed no significant differences at all. Over one-half of pharmacists (55.9%) worked in the retail sector. Proportionately more male than female pharmacists in both employment categories and proportionately more part-time than full-time pharmacists of both genders worked in this sector.

### 3.2. Estimated Equations

The values of the estimated least-squares coefficients, their standard errors, and (two-tail) levels of statistical significance are presented in Table 2. The *F* ratios of all four equations were significant and the relatively high adjusted R^2^ values suggested that the results were robust.

The coefficients of both covariates for work input behaved as expected and were highly significant throughout the equations. At the respective employment-category and gender means of the variable, an additional 100 h of work input yielded income increases of 3.8% for male full-time pharmacists, 3.9% for female full-time pharmacists, 17.1% for male part-time pharmacists, and 13.7% for female part-time pharmacists.

The coefficients for age as a proxy for experience also behaved as expected and were statistically significant in all four equations. The estimated parameters suggested that, at the means of the variable, an additional year of experience resulted in annual income going up by 0.7% for male full-time pharmacists, 0.8% for female full-time pharmacists, 3.0% for male part-time pharmacists, and 1.5% for female part-time pharmacists.

The non-married covariate’s coefficients were negative and significant except for male part-time pharmacists. The findings showed that the unmarried penalty, in terms of less income earned compared to their married counterparts, was 7.0% for male full-time pharmacists, 12.3% for female full-time pharmacists, and 36.7% for female part-time pharmacists.

The coefficients for being Black/African American were significant for both genders of full-time pharmacists, but not significant for part-time pharmacists. Black/African American men who worked full-time earned, on average, 15.1% less than non-Black/African American male, full-time pharmacists, while Black/African American women who worked full-time earned 10.2% less than their non-Black/African American counterparts. Regarding Hispanic pharmacists, all four coefficients were statistically significant and negative, meaning that Hispanics consistently earned less income than pharmacists who were not Hispanic: male full-time pharmacists, 6.8%; female full-time pharmacists, 13.8%; male part-time pharmacists, 39.4%; and female part-time pharmacists, 24.6%.

Finally, the estimated coefficients for pharmacists who worked in the retail sector were significant for three of the four categories (only part-time, male pharmacists failed to show significance) but did not behave as expected. They were negative, meaning that retail pharmacists earned less income than their peers in their respective categories who worked in hospitals, manufacturing, or other settings. Male full-time pharmacists earned 3.4% less, female full-time pharmacists earned 4.0% less, and female part-time pharmacists earned 15.4% less.

## 4. Discussion

Some of the estimated values reported in the previous section are subject to further analysis to avoid biased interpretations. For example, part of the reason why male full-time pharmacists earned higher income levels than female full-time pharmacists was that they worked, on average, more hours. Similarly, female part-time pharmacists earned higher income levels than their male part-time counterparts partly because they worked relatively more hours. An average wage rate for each of the four groups, obtained by dividing the average number of hours worked per year into average annual income earned, would remove the biases from the intergroup comparisons. The calculated average wage rate values, $66.36 for male full-time pharmacists, $61.78 for female full-time pharmacists, $60.68 for male part-time pharmacists, and $64.27 for female part-time pharmacists, confirmed that indeed male full-time pharmacists were better paid than female full-time pharmacists and female part-time pharmacists were better paid than male part-time pharmacists. The observed income differences between male and female pharmacists, especially within full-time and part-time categories, are aligned with overall developments in the healthcare sector. Research indicates that women in pharmacy often face challenges such as limited access to ownership opportunities and leadership roles, which can impact earnings potential. Additionally, women in pharmacy may encounter barriers to mentorship and professional networking, further hindering career advancement and equitable compensation [30,31,32].

Another empirical finding that might lead to a biased interpretation is that the percentage increase in income out of an additional 100 h of work is much greater for part-time than full-time pharmacists of both genders. Obviously an additional 100 h of work constitutes a greater proportion of time for part-time workers, so it is not surprising to observe a greater income percentage rise that goes along with it. What is needed to remove the bias is to calculate the work-input elasticities of earnings. This indicator represents the ratio of the percentage change in income resulting from a percentage increase in hours worked. The elasticity values (see Table 3), computed at the means of the covariates, showed that a 10% increase in the number of hours worked indeed had a greater impact on part-time than full-time pharmacists of both genders. These results are consistent with findings by Carvajal and Popovici (2016) [15], who analyzed survey data from pharmacists in South Florida and found that a 10% longer workweek raised earnings by 14.6% for part-timers compared to 2.8% for full-timers. For comparison purposes the age elasticity of earnings values also were calculated and presented in Table 3. These values (at the means of the covariates) revealed that age was more earnings elastic for part-time than full-time pharmacists of both genders as well.

Three major generalizations may be drawn from the investigation into the full-time and part-time pharmacists’ income determination process conducted here. First, pharmacists who worked full-time were substantially different in several ways from their peers who worked part-time. Part-time work was selective of women, and on average it amounted to slightly more than one-third of the average full-time work input. Significant differences in marital status and percentage working in retail pharmacy were detected for both genders, and female part-time pharmacists were older and recorded a lower percentage of Blacks/African Americans than full-time pharmacists.

Pharmacists also responded differently to some of the identical socioeconomic stimuli. With respect to the least-squares coefficients, part-time pharmacists’ estimated constant terms were lower than those of full-time pharmacists, which lends credence to the contention by Carvajal and Popovici [15] that pharmacists who work fewer hours are driven almost exclusively by pay, whereas pharmacists working more hours tend to exhibit a more comprehensive approach to income determination. This conclusion was further supported by the finding that all coefficients for full-time pharmacists (both genders) were statistically significant, but several of the part-time pharmacists’ coefficients lacked significance. Furthermore, as already pointed out, both work input and age were more earnings elastic among part-time than full-time pharmacists and female part-time pharmacists were more heavily penalized, in terms of earnings, than either gender of full-time pharmacists for not being married or working in the retail sector (the coefficients for male part-time pharmacists lacked significance). In addition, both coefficients for part-time Black/African American pharmacists lacked significance, while the full-time Black/African American pharmacists’ coefficients were highly significant, and the Hispanic income gap for both genders was greater for part-time than full-time pharmacists.

The second generalization from this study is that gender played a key role in shaping the full-time versus part-time comparison profile. Almost four out of every five (79.4%) part-time pharmacists were women. Within full-time pharmacists, men earned more income, worked more hours, and exhibited a higher wage rate than women, but within part-time pharmacists, women earned more income, worked more hours, and showed a higher wage rate than men. Within full-time pharmacists, men were generally older and proportionately fewer men than women reported not being married and being Black/African American, whereas within part-time pharmacists, no significant difference in age was detected and proportionately more men than women reported not being married and being Black/African American. Working in retail pharmacy was the only variable in which the gender relationship—in this case more men than women—accorded for both employment categories.

Additional gender comparisons revealed that part-time men’s income was more work-input and age elastic than part-time women’s income, although no gender differences were found within full-time pharmacists. Gender comparisons of the effect of the other covariates on income were flawed by the absence of statistical significance in some of the coefficients for male part-time pharmacists. Compared to their respective full-time pharmacists who were married, female pharmacists were penalized more than their male peers for not being married, but not as much as female part-time pharmacists. Black/African American men who worked full-time earned proportionately less than their Black/African American women counterparts. (No significant difference was found within part-time pharmacists.) Hispanic women who worked full-time earned proportionately less than Hispanic men who worked full-time, but within part-time pharmacists, Hispanic men were penalized more than Hispanic women. Finally, female part-time pharmacists who worked in the retail sector were paid proportionately less than their full-time peers of both genders.

Third, the empirical results obtained here point toward the existence of major income gaps affected by race, ethnic group, marital status, and type of practice. In terms of the overall population, Blacks/African Americans and Hispanics were underrepresented in the pharmacist workforce, each accounting for less than 5%. They also were paid, on average, substantially less than nonminority pharmacists—full-time Black/African American men and women earned 15.1% and 10.2% less, respectively, than their full-time peers who were not Black/African American (the part-time coefficients were not significant), while Hispanic pharmacists earned less income across the board; Hispanic part-time pharmacists were much more penalized income wise (39.4% less for men and 24.6% less for women) than full-time pharmacists (6.8% men and 13.8% less for women). These disparities are not isolated but reflect long-standing patterns of wage discrimination in the healthcare industry. Historical factors, including occupational segregation and discriminatory hiring practices, have contributed to the underrepresentation and undervaluation of minority pharmacists. For example, Black and Hispanic women in healthcare have historically earned significantly less than their White counterparts, with Black women earning 63 cents and Hispanic women earning 58 cents for every dollar earned by White, non-Hispanic men. Moreover, systemic barriers in education, hiring, and promotion continue to affect the earnings potential of minority pharmacists [33,34].

The income gap for pharmacists who were not married also was substantial, especially for female part-time practitioners, who earned on average 36.7% less than their married counterparts. Finally, retail pharmacists also experienced an income gap, earning less, other things equal, than pharmacists who worked in other types of practice.

## 5. Limitations

Empirical studies have limitations, and the current probe into the income determination process by employment category and gender is no exception. This study relied on self-reported survey data, which always are subject to validity and reliability concerns. Answers in self-reported data are influenced by subjective feelings and perceptions that sometimes blur the true roots of income disparities due to gender and employment category. Moreover, neither the accuracy of the responses was measurable, nor the identity of the respondents was verifiable.

Another limitation is that the 2019–2022 data used here might not have been entirely homogeneous. The 2019–2022 data may not fully reflect typical pharmacist behavior, as this period included the COVID-19 pandemic. The pandemic affected wages, employment status, and work hours, with increased demand in hospitals and clinical settings, mixed impacts in retail, and elevated burnout leading some pharmacists to leave or change roles. As a result, the patterns observed in earnings and work input during this period may differ from long-term trends. Also, pharmacy-related earnings were uniformly adjusted to 2022 dollars using the CPI, a method that may be somewhat arbitrary and could overlook important individual variations within the study period. This approach might not account for annual income fluctuations within the 2019–2021 pharmacist samples.

A third limitation is that the dependent variable in the estimated equations was nominal income, that is, the value reported by practitioners in the ACS. This variable did not take into consideration spatial variations in taxes and cost of living, which are essential in configuring real income. Since real income usually guides socioeconomic behavior more precisely than nominal income, the statistical significance of the estimated coefficients and goodness-of-fit values might have improved if real income had been used as the dependent variable.

Still, another limitation is that this study focused solely on the 2019–2022 analysis period. Thus, the setting was inadequate to assess whether employment category- and gender-related income disparities evolve or the way in which the various determinants influence income over time. In addition, a potential bias might have occurred from the omission of relevant covariates; while the covariates appearing in the equations are frequently identified in the literature, the possibility remains that others might have been overlooked.

## 6. Conclusions

Despite its limitations, this study has served as a vehicle for probing the nature and magnitude of differences between U.S. full-time and part-time pharmacists and has established that they vary substantially in their composition and response to identical socioeconomic stimuli in the income determination process. It also has pointed out the influence of gender in shaping these differences, as well as the existence of substantial income gaps affected by race, ethnic group, marital status, and practice site.

The findings reported here have important implications for scholars and workforce managers. Understanding differences between full-time and part-time pharmacists is important because employment status shapes earnings, career opportunities, and workforce retention. These patterns also have implications for equity within the profession and for ensuring adequate access to pharmacy services in communities. They have corroborated previous findings and hopefully will serve as a steppingstone for future research to provide guidelines that enhance practitioners’ hiring, productivity, and retention.

## Figures and Tables

**Table 1:** Means and standard deviations (in parentheses) of variables in the income determination model of full-time and part-time pharmacists.

	Full-Time Pharmacists		Part-Time Pharmacists	
Variable	Men	Women	Men	Women
Number of observations	4387	6318	280	1079
				
Annual income (2022 dollars)	141,300 ^1a^	126,978 ^1a^	44,221 ^1a^	53,687 ^1a^
	(65,013)	(49,877)	(42,138)	(34,138)
				
Work input (hours per year)	2129.4 ^1a^	2055.2 ^1a^	728.8 ^1a^	835.3 ^1a^
	(453.0)	(438.0)	(402.7)	(406.8)
				
Age (years)	42.8 ^1^	41.0 ^1a^	43.0	43.5 ^a^
	(11.0)	(10.4)	(15.0)	(11.4)
				
Currently not married (%)	0.279 ^1a^	0.309 ^1a^	0.443 ^1a^	0.215 ^1a^
	(0.448)	(0.462)	(0.498)	(0.411)
				
Black/African American (%)	0.045 ^2^	0.055 ^2a^	0.061 ^2^	0.029 ^2a^
	(0.208)	(0.228)	(0.239)	(0.167)
				
Hispanic (%)	0.045	0.042	0.064	0.036
	(0.207)	(0.201)	(0.246)	(0.187)
				
Working in retail pharmacy (%)	0.580 ^1a^	0.525 ^1a^	0.736 ^1a^	0.627 ^1a^
	(0.494)	(0.499)	(0.442)	(0.484)

^1^ Gender differences within employment category were statistically significant (ρ ≤ 0.01). ^2^ Gender differences within employment category were statistically significant (ρ ≤ 0.05). ^a^ Employment-category differences within gender were statistically significant (ρ ≤ 0.01).

**Table 2:** Estimated least-squares coefficients, their standard errors (in parentheses), and (two-tail) levels of significance of covariates in the income determination model of full-time and part-time pharmacists.

		Full-Time Pharmacists (*i* = 1)		Part-Time Pharmacists (*i* = 2)	
Covariate	Term	Men (*j* = 1)	Women (*j* = 2)	Men (*j* = 1)	Women (*j* = 2)
					
Constant term	α	8.838925	8.830065	4.989611	6.716941
					
Work input	λ_1_	0.120213 *	0.127941 *	0.290659 *	0.333445 *
		(0.004377)	(0.003503)	(0.045424)	(0.020456)
					
Work input squared	λ_2_	−0.001911 *	−0.002128 *	−0.008540 *	−0.011573 *
		(0.000089)	(0.000077)	(0.002874)	(0.001295)
					
Age	γ_1_	0.057151 *	0.053106 *	0.157305 *	0.093152 *
		(0.005277)	(0.004306)	(0.030674)	(0.013979)
					
Age squared	γ_2_	−0.000577 *	−0.000545 *	−0.001466 *	−0.000891 *
		(0.000059)	(0.000050)	(0.000340)	(0.000156)
					
Currently not married	θ_1_	−0.067481 *	−0.115560 *	−0.034640	−0.312802 *
		(0.014756)	(0.011370)	(0.090439)	(0.046543)
					
Black/African American	θ_2_	−0.140986 *	−0.096722 *	−0.001815	−0.137478
		(0.024728)	(0.019250)	(0.136179)	(0.078588)
					
Hispanic	θ_3_	−0.065730 ^†^	−0.129653 *	−0.332499 ^†^	−0.219621 ^†^
		(0.027866)	(0.023968)	(0.139367)	(0.092383)
					
Work in retail pharmacy	ϕ	−0.033852 *	−0.039603 *	0.035754	−0.143436 *
		(0.012604)	(0.010261)	(0.086367)	(0.035523)
					
F statistic		188.1*	303.3 *	64.6 *	278.2 *
				
Adjusted R^2^		0.254	0.277	0.646	0.673

* Statistically significant (ρ ≤ 0.01). ^†^ Statistically significant (ρ ≤ 0.05).

**Table 3:** Income elasticities by employment category and gender calculated at the means of the covariates from the estimated coefficients shown in Table 2.

	Full-Time Pharmacists		Part-Time Pharmacists	
				
Variable	Men	Women	Men	Women
Work input	0.77	0.77	1.24	1.15
				
Age	0.23	0.26	1.13	0.52

## Data Availability

Data supporting the results of this study can be found at https://usa.ipums.org/usa/ (accessed on 1 May 2024).
